# The Case for Angioplasty in Patients with Symptomatic Intracranial Atherosclerosis

**DOI:** 10.3389/fneur.2014.00036

**Published:** 2014-04-11

**Authors:** Ryan A. McTaggart, Michael P. Marks

**Affiliations:** ^1^Department of Radiology, Cleveland Clinic Florida, Weston, FL, USA; ^2^Department of Neurosurgery, Cleveland Clinic Florida, Weston, FL, USA; ^3^Cerebrovascular Center, Neurological Institute, Cleveland Clinic, Cleveland OH, USA; ^4^Department of Radiology, Stanford University Medical Center, Stanford, CA, USA; ^5^Department of Neurosurgery, Stanford University Medical Center, Stanford, CA, USA

**Keywords:** angioplasty, stenosis, stents, intracranial stenosis, intracranial atherosclerosis

## Abstract

Intracranial atherosclerotic disease (ICAD) is likely the most common cause of stroke worldwide and remains highly morbid even with highly monitored medical therapy. Recent results of the SAMMPRIS trial, which randomized patients to stenting plus aggressive medical management versus aggressive medical management alone have shown that additional treatment of intracranial atherosclerotic lesions with the Wingspan stent is inferior to aggressive medical management alone. In light of these results, there has been renewed interest in angioplasty alone to treat symptomatic ICAD. This article will briefly review the natural history of ICAD and discuss the possible future for endovascular treatment of ICAD with primary intracranial angioplasty in appropriately selected patients.

## Epidemiology and Natural History

Several important natural history and medical treatment studies have been published that allow us to appreciate the impact of ICAD (1–4). Approximately 5–10% of all strokes and TIA’s are due to ICAD (5). A landmark, prospective multi-center study of 4157 patients admitted within 24 h of ischemic onset demonstrated symptomatic intracranial stenosis ( >50%) in 6.5% of patients (6). The study showed proximal middle cerebral artery and basilar artery occlusions were seen in 3.7 and 1.2%, respectively (6). Mortality rates at 100 days were highest in the basilar artery occlusion group (44.7%) and were and 10.1 and 21.4% in the symptomatic intracranial atherosclerosis ( >50%) and middle cerebral artery occlusion groups, respectively. The incidence of intracranial atherosclerosis does vary by race and is more prevalent in Chinese, Japanese, African-American, and Hispanic patients (7, 8), as compared with elevated extracranial atherosclerosis rates in white patients (9, 10).

The greatest concern for ICAD patients, particularly those with ≥70% stenosis, is the risk of subsequent stroke. While some medical subgroup data suggesting 7–8% stroke rates in untreated patients with symptomatic stenosis are available from the 1980s (11), the data are limited by selection bias and inadequate follow-up. Later studies aimed to address stroke risk (2, 12, 13). In a cohort of 705 Chinese patients who presented with acute ischemic stroke, Wong et al. reported 1-year stroke rate of 17.1% in patients with intracranial atherosclerosis only and 24.3% in patients in patients with both intracranial and cervical disease. Even more sobering data were derived in a study by Asil et al. (13) where 13 of 38 (38%) patients with >50% stenosis who completed 6-month follow-up had a stroke. Finally, in a study of patients with >50% stenosis, the GESICA (Groupe d’Etude des Stenoses Inta-Cranieenes Atheromateuses symptomatiques) study (2) patients had a 38.2% rate of a cerebrovascular event during approximately 2 years of follow-up, in spite of antiplatelet or anticoagulant therapy.

Our best natural history data for ICAD prior to the publication of the SAMMPRIS trial (4) came from the prospective, randomized Warfarin-Aspirin Symptomatic Intracranial Disease (WASID) trial (1). This prospective, multi-center study randomized patients to ASA or warfarin. To be included, patients had to have symptomatic ICAD ( >50% narrowing) and the 2-year primary endpoints were ischemic stroke, brain hemorrhage, and death from vascular causes. While WASID was criticized for its non-standard ASA regimen and high rate of dropout for both medications, warfarin offered no benefit over aspirin in preventing recurrent stroke, and the primary endpoints were reached in 21% in the aspirin group and 22% in the warfarin group. Notably, patients in the warfarin arm of the study had significantly higher rates of hemorrhage and for this reason and the lack of efficacy WASID was terminated prematurely.

SAMMPRIS targeted a group of high-risk, patients identified in subgroup analyses of WASID (14–16). This high-risk subgroup (14) demonstrated a 23% stroke risk at 12 months and was composed of patients with severe stenosis ( >70%) enrolled earlier than 17 days after symptom onset. Another subgroup analyses (15, 16) germane to the construction of SAMMPRIS was the identification of elevated blood pressure and cholesterol levels as predictive of future stroke and other vascular events in symptomatic patients with intracranial stenosis and were the basis of the aggressive medical management used for both arms of SAMPRISS.

The SAMMPRIS trial (4) randomized patients with symptomatic intracranial stenosis ( ≥70%) to aggressive medical management versus endovascular therapy with aggressive medical management. Enrollment in the trial was halted after 451 patients underwent randomization because the 30-day stroke and death rate in the group receiving endovascular therapy and medical management was 14.7% versus 5.8% in the group receiving medical management alone. In addition, the probability of experiencing the primary end point (any stroke or death within 30 days after enrollment or after any revascularization procedure of a qualifying lesion or a stroke in the territory of the symptomatic artery beyond 30 days) at 1-year was 12.2% in the medical management group.

It is important to recognize, as stated above, that the importance of aggressive medical management was derived from the WASID data (15, 16) and ultimately tested in SAMMPRIS. Aggressive medical management (available to both arms of the SAMMPRIS trial) consisted of two anti-platelet agents, a statin (goal LDL < 70), and one medication from each major class of antihypertensive agents (goal SBP < 140; 130 if diabetic). Patient compliance and risk factor management were managed at each site by a team including a neurologist, a study coordinator, and a lifestyle coach (17). Compliance with medical regimens was closely monitored by the study coordinator including counting patients’ anti-platelet medications. The lifestyle coach met with the patients to develop personal action plans and contacted the patients every 2 weeks for the first 3 months and then monthly thereafter. Additional help was provided for difficult-to-manage patients from a central director.

There is little question that aggressive medical management of ICAD has a profound effect on the natural history of the disease. While we should try to achieve the medical management parameters set forth in SAMMPRIS, this degree of oversight is costly and it is quite likely that medical management applied long-term to “real-world” situations might result in event rates for symptomatic intracranial stenosis that were higher than those seen in SAMMPRIS. In addition, it should be pointed out that the “low” event rate in SAMMPRIS left more than 1 in 10 patients with a death or a stroke in the territory of the symptomatic artery at 1 year. The endovascular comparator in SAMMPRIS was stent placement with a self-expanding stent and this clearly raises the question whether another endovascular strategy such as angioplasty alone may provide better results.

## Intracranial Angioplasty

Angioplasty used in the setting of symptomatic intracranial stenosis has been performed for more than two decades and was motivated by the poor natural history of ICAD despite medical therapy. While the first successful intracranial angioplasty was reported by Thoralf Sundt in 1980, early results showed high complication rates. For example, Higashida et al. treated eight symptomatic patients and encountered three major complications (38%) (18).

Subsequent technical advances reduced these early complications. One very important contribution to the safety of balloon angioplasty was the concept of “sub-maximal angioplasty” first promoted by Connors et al. (19). They examined three time periods in their angioplasty experience based on the technique used. In early experience, the angioplasty balloon size approximated the vessel size with rapid angioplasty. In the middle experience over-sizing of the balloon was permitted with rapid angioplasty. In the final time period, an under-sized balloon was used with slow inflations. Numbers in the groups were small and both variables (inflation times and balloon size) were not controlled for. Nevertheless, these data indicated procedural complications rates may be reduced by slow expansion of a balloon smaller than the native artery.

Table 1 reports the technical success of several groups (19–26) and suggests the 30-day major complication rates are ≤6%. Many of these same investigators also show low post-procedure stroke rates over time and this ultimately drives the case for angioplasty in patients with symptomatic intracranial atherosclerosis (Table 2).

**Table 1:** **Technical success and 30-day major complications of angioplasty**.

Series	*N* (cohort size)	Complication rate (%)	Technical success (%)
Higashida et al. (18)	8	38	
Clark et al. (20)	17	9.1	
Marks et al. (21)	23	4.3	
Connors et al. (19)	50	6	98
Yoon et al. (22)	32	6	91
Marks et al. (23)	120	5.8	93
Wojak et al. (24)	60	4.8	91
Nguyen et al. (25)	74	5.0	92
Dumont et al. (26)	41	4.9	

**Table 2:** **Long-term stroke rates following angioplasty**.

Series	*N* (cohort size)	Mean follow-up (months)	Annual stroke rate (%)
Clark et al. (20)	17	22	0
Yoon et al. (22)	32	20	0
Marks et al. (23)	120	42	3.2
Wojak et al. (24)	60	45	1.8
Nguyen et al. (25)	74	3	N.R.
Dumont et al. (26)	41	12	3.1

Clark and Yoon demonstrated notable results in studies of 17 and 32 patients, respectively (20, 22) with the latter study reporting a single TIA event (Table 2). The two largest series – Marks et al. (23) and Wojak et al. (24) – also showed favorable stroke rates. Marks et al. had a 3.2% rate of death and stroke in the territory corresponding to treatment (42-month follow-up) and Wojak et al. had an annual stroke rate of 1.8% (45-month follow-up). Although their follow-up was only 3 months and no annual stroke rate could be reported, Nguyen et al. (25) had only one stroke in the territory ipsilateral to the treated vessel. However, four major procedure-related strokes occurred in this multi-center study.

In a study published in response to SAMPRISS, Dumont et al. (26) queried their database of 41 patients [many of whom were ineligible for SAMPRISS and Vitesse Intracranial Stent Study for Ischemic Therapy (VISSIT)] who underwent intracranial sub-maximal balloon angioplasty procedures between January 2007 and July 2011. These patients had >70% stenosis and many presented with an acute ischemic event. In 32 patients with at least 12 months of follow-up, only 1 ischemic event (a TIA) in the vascular distribution of the treated vessel occurred between 30 days and 1-year after the procedure. However, the 1-year event-free survival rate was 91% (29 of 32 patients) as two patients had peri-procedural morbidity.

It cannot escape attention that the angioplasty studies show substantially lower 30-day peri-procedural complications when compared to stent treatment arm of the SAMMPRIS study. In addition, the post-treatment annual stroke rates discussed above are superior to the natural history reported in those patients treated with aggressive medical therapy in SAMMPRIS. However, randomized data comparing a strategy of primary angioplasty with best medical management alone are lacking.

## Patient Selection and Technique

In our current post-SAMMPRIS practice, we generally follow three tenets when applying angioplasty to the treatment of symptomatic ICAD. The patient should fail best medical therapy (dual antiplatelet, statins, and risk factor reduction). The operator must use sub-maximal angioplasty technique. The operator should only select those lesions likely to respond to balloon dilatation. SAMPRISS demonstrated the profound impact of best medical therapy and we have discussed sub-maximal angioplasty technique. Perhaps equally important is the issue of lesion selection. Failure to appreciate the impact of lesion morphology or employ sub-maximal angioplasty technique would be a serious oversight by the endovascular surgeon.

Mori et al. (27) hypothesized lesion morphology would affect lesion response to angioplasty and thus categorized atherosclerotic lesions as short, concentric and <5 mm long (Mori A), 5–10 mm long and may be eccentric (Mori B), and >10 mm and may have excessive tortuosity (Mori C). As predicted, Mori found higher rates of death, ipsilateral stroke, or subsequent ipsilateral bypass after angioplasty by lesion type; Type A (8%) versus Type B (26%) versus Type C (87%). While two studies found no outcome differences between lesions >7 or <7 mm (28, 29), a number of other studies support the Mori data and have found lesion length or morphology an important variable in determining procedural success and restenosis rates (30–34).

## The Future of Intracranial Angioplasty

Despite the low 1 year stroke rates following intracranial angioplasty, restenosis remains a possible weakness of primary angioplasty. Symptomatic and angiographic restenosis (23, 24, 26) occur at 6 months in approximately 5–30% of patients treated with angioplasty alone. The re-angioplasty rate was in excess of 20% in the most recent study in which sub-maximal technique was rigorously employed.

The drug-eluting balloon (DEB) may alter this problem. The DEB is an emerging technology with meaningful accumulated data in coronary arteries and femoropopliteal disease. The sina qua non-for this technology is effective transfer, absorption, and circumferentially uniform effect of the drug on the diseased vessel segment during the short period the balloon is inflated against an atherosclerotic plaque. Excipient technology (the balloon coating that helps deliver the drug) is in its infancy and one should expect a plethora of proprietary formulas in years to come. Paclitaxel is favored as it is highly lipophilic and allows for passive absorption through cell membranes with sustained effect within a treated vessel wall.

Data regarding the use of DEB’s in the small vessels to which we are accustomed are scant. In a single-arm study, Schmidt et al. (35) treated patients with infra-popliteal disease and 70% stenosis with a paclitaxel-eluting balloon (In.Pact Amphirion, Medtronic, Minneapolis, MN, USA) with pre-dilatation and 1 min inflation times. The 3-month restenosis rate (available for 84 of the 109 limbs treated) was 27.4%, which compares quite favorably with the 60–70% restenosis rate typically seen with uncoated balloons. There have been two reports by one endovascular group of DEB use for intracranial atherosclerotic lesions (36, 37). In their first report, they compared DEB and conventional angioplasty balloons for the treatment of in-stent recurrent stenosis and found DEB’s reduced subsequent restenosis fivefold (9 versus 50%) (36). In their most recent report, 52 patients with high-grade ICAD lesions underwent primary angioplasty and stenting with the Enterprise stent. Angioplasty with the DEB was performed in >80% of patients and in 33 patients with an average follow-up of 8.9 months only 1 (3%) recurrent stenosis was seen (37).

A possible dilemma for DEB intracranial angioplasty will be the need to balance “sub-maximal angioplasty” (slow inflation of under-sized balloons) with effective circumferential coating of the diseased vessel with excipient and drug. Furthermore, the risk and consequence of embolization of excipient and drug to intracranial branches beyond the ICAD lesion is, at present, unknown.

## Conclusion

The first line of therapy for symptomatic ICAD patients is dual anti-platelet therapy and aggressive management of blood pressure, blood sugar, and lipids. The SAMMPRIS trial gave us useful information in that even with aggressive, highly monitored medical management, there is still a concerning 12.2% combined 30-day stroke and death rate or ipsilateral stroke rate beyond 30 days in the first year of treatment. The results of the SAMMPRIS trial should not stop further investigation of endovascular therapy for severe symptomatic intracranial stenosis. With appropriate lesion selection and technique, intracranial angioplasty should be a technically safe procedure with a low complication rate. Furthermore, single center series suggests there is a low annual stroke rate in these patients. However, no randomized data exists to show a benefit compared to medical therapy. The impact of DEB’s on post-treatment stroke rates and restenosis rates is eagerly awaited.

## Conflict of Interest Statement

The authors declare that the research was conducted in the absence of any commercial or financial relationships that could be construed as a potential conflict of interest.

## References

[1] Chimowitz MI Lynn MJ Howlett-Smith H Stern BJ Hertzberg VS Frankel MR Comparison of warfarin and aspirin for symptomatic intracranial arterial stenosis. N Engl J Med (2005) 352:1305–16 10.1056/NEJMoa043033 15800226

[2] Mazighi M Tanasescu R Ducrocq X Vicaut E Bracard S Houdart E Prospective study of symptomatic atherothrombotic intracranial stenoses: the GESICA study. Neurology (2006) 66:1187–91 10.1212/01.wnl.0000208404.94585.b2 16636236

[3] Gorelick PB Wong KS Bae HJ Pandey DK Large artery intracranial occlusive disease: a large worldwide burden but a relatively neglected frontier. Stroke (2008) 39:2396–9 10.1161/STROKEAHA.107.505776 18535283

[4] Chimowitz MI Lynn MJ Derdeyn CP Turan TN Fiorella D Lane BF Stenting versus aggressive medical therapy for intracranial arterial stenosis. N Engl J Med (2011) 365:993–1003 10.1056/NEJMoa1105335 21899409 PMC3552515

[5] Wityk RJ Lehman D Klag M Coresh J Ahn H Litt B Race and sex differences in the distribution of cerebral atherosclerosis. Stroke (1996) 27:1974–80 10.1161/01.STR.27.11.1974 8898801

[6] Weimar C Goertler M Harms L Diener HC Distribution and outcome of symptomatic stenoses and occlusions in patients with acute cerebral ischemia. Arch Neurol (2006) 63:1287–91 10.1001/archneur.63.9.1287 16966507

[7] Feldmann E Daneault N Kwan E Ho KJ Pessin MS Langenberg P Chinese-white differences in the distribution of occlusive cerebrovascular disease. Neurology (1990) 40:1541–5 10.1212/WNL.40.10.1540 2215945

[8] Sacco RL Roberts JK Boden-Albala B Gu Q Lin IF Kargman DE Race-ethnicity and determinants of carotid atherosclerosis in a multiethnic population. The Northern Manhattan Stroke Study. Stroke (1997) 28:929–35 10.1161/01.STR.28.5.929 9158627

[9] Heyden S Heyman A Goree JA Nonembolic occlusion of the middle cerebral and carotid arteries – a comparison of predisposing factors. Stroke (1970) 1:363–9 10.1161/01.STR.1.5.363 5522932

[10] Fields WS Lemak NA Joint study of extracranial arterial occlusion. X. Internal carotid artery occlusion. JAMA (1976) 235:2734–8 10.1001/jama.235.24.2608 946886

[11] Group T.E.I.B.S. Failure of extracranial-intracranial arterial bypass to reduce the risk of ischemic stroke. Results of an international randomized trial. The EC/IC Bypass Study Group. N Engl J Med (1985) 313:1191–200 10.1056/NEJM198511073131904 2865674

[12] Wong KS Li H Long-term mortality and recurrent stroke risk among Chinese stroke patients with predominant intracranial atherosclerosis. Stroke (2003) 34:2361–6 10.1161/01.STR.0000089017.90037.7A 12947158

[13] Asil T Balci K Uzunca I Kerimoglu M Utku U Six-month follow-up study in patients with symptomatic intracranial arterial stenosis. J Clin Neurosci (2006) 13:913–6 10.1016/j.jocn.2006.01.043 17049246

[14] Kasner SE Chimowitz MI Lynn MJ Howlett-Smith H Stern BJ Hertzberg VS Predictors of ischemic stroke in the territory of a symptomatic intracranial arterial stenosis. Circulation (2006) 113:555–63 10.1161/CIRCULATIONAHA.105.578229 16432056

[15] Chaturvedi S Turan TN Lynn MJ Kasner SE Romano J Cotsonis G Risk factor status and vascular events in patients with symptomatic intracranial stenosis. Neurology (2007) 69:2063–8 10.1212/01.wnl.0000279338.18776.26 18040012

[16] Turan TN Cotsonis G Lynn MJ Chaturvedi S Chimowitz M Warfarin-Aspirin Symptomatic Intracranial Disease Trial Relationship between blood pressure and stroke recurrence in patients with intracranial arterial stenosis. Circulation (2007) 115:2969–75 10.1161/CIRCULATIONAHA.106.622464 17515467

[17] Chimowitz MI Lynn MJ Turan TN Fiorella D Lane BF Janis S Design of the stenting and aggressive medical management for preventing recurrent stroke in intracranial stenosis trial. J Stroke Cerebrovasc Dis (2011) 20:357–68 10.1016/j.jstrokecerebrovasdis.2011.05.001 21729789 PMC3506385

[18] Higashida RT Tsai FY Halbach VV Dowd CF Smith T Fraser K Transluminal angioplasty for atherosclerotic disease of the vertebral and basilar arteries. J Neurosurg (1993) 78:192–8 10.3171/jns.1993.78.2.0192 8421202

[19] Connors JJ III Wojak JC Percutaneous transluminal angioplasty for intracranial atherosclerotic lesions: evolution of technique and short-term results. J Neurosurg (1999) 91:415–23 10.3171/jns.1999.91.3.0415 10470816

[20] Clark WM Barnwell SL Nesbit G O’Neill OR Wynn ML Coull BM Safety and efficacy of percutaneous transluminal angioplasty for intracranial atherosclerotic stenosis. Stroke (1995) 26:1200–4 10.1161/01.STR.26.7.1200 7604414

[21] Marks MP Marcellus M Norbash AM Steinberg GK Tong D Albers GW Outcome of angioplasty for atherosclerotic intracranial stenosis. Stroke (1999) 30:1065–9 10.1161/01.STR.30.5.1065 10229745

[22] Yoon W Seo JJ Cho KH Kim MK Kim BC Park MS Symptomatic middle cerebral artery stenosis treated with intracranial angioplasty: experience in 32 patients. Radiology (2005) 237:620–6 10.1148/radiol.2372041620 16192322

[23] Marks MP Wojak JC Al-Ali F Jayaraman M Marcellus ML Connors JJ Angioplasty for symptomatic intracranial stenosis: clinical outcome. Stroke (2006) 37:1016–20 10.1161/01.STR.0000206142.03677.c2 16497979

[24] Wojak JC Dunlap DC Hargrave KR Dealvare LA Culbertson HS Connors JJ III Intracranial angioplasty and stenting: long-term results from a single center. AJNR Am J Neuroradiol (2006) 27:1882–92 17032860 PMC7977880

[25] Nguyen TN Zaidat OO Gupta R Nogueira RG Tariq N Kalia JS Balloon angioplasty for intracranial atherosclerotic disease: periprocedural risks and short-term outcomes in a multicenter study. Stroke (2011) 42:107–11 10.1161/STROKEAHA.110.583245 21071722

[26] Dumont TM Kan P Snyder KV Hopkins LN Siddiqui AH Levy EI Revisiting angioplasty without stenting for symptomatic intracranial atherosclerotic stenosis after the stenting and aggressive medical management for preventing recurrent stroke in intracranial stenosis (SAMMPRIS) study. Neurosurgery (2012) 71:1103–10 10.1227/NEU.0b013e318271bcb8 22986593

[27] Mori T Fukuoka M Kazita K Mori K Follow-up study after intracranial percutaneous transluminal cerebral balloon angioplasty. AJNR Am J Neuroradiol (1998) 19:1525–33 9763389 PMC8338699

[28] Suh DC Kim JK Choi JW Choi BS Pyun HW Choi YJ Intracranial stenting of severe symptomatic intracranial stenosis: results of 100 consecutive patients. AJNR Am J Neuroradiol (2008) 29:781–5 10.3174/ajnr.A0922 18310234 PMC7978185

[29] Qureshi AI Tariq N Hassan AE Vazquez G Hussein HM Suri MF Predictors and timing of neurological complications following intracranial angioplasty and/or stent placement. Neurosurgery (2011) 68:53–60; discussion 60–61. 10.1227/NEU.0b013e3181fc5f0a 21150755

[30] Miao ZR Feng L Li S Zhu F Ji X Jiao L Treatment of symptomatic middle cerebral artery stenosis with balloon-mounted stents: long-term follow-up at a single center. Neurosurgery (2009) 64:79–84; discussion 84–75. 10.1227/01.NEU.0000335648.31874.37 19050657

[31] Kurre W Berkefeld J Brassel F Bruning R Eckert B Kamek S In-hospital complication rates after stent treatment of 388 symptomatic intracranial stenoses: results from the INTRASTENT multicentric registry. Stroke (2010) 41:494–8 10.1161/STROKEAHA.109.568063 20075358

[32] Zhu SG Zhang RL Liu WH Yin Q Zhou ZM Zhu WS Predictive factors for in-stent restenosis after balloon-mounted stent placement for symptomatic intracranial atherosclerosis. Eur J Vasc Endovasc Surg (2010) 40:499–506 10.1016/j.ejvs.2010.05.007 20554461

[33] Al-Ali F Cree T Hall S Louis S Major K Smoker S Predictors of unfavorable outcome in intracranial angioplasty and stenting in a single-center comparison: results from the Borgess Medical Center-Intracranial Revascularization Registry. AJNR Am J Neuroradiol (2011) 32:1221–6 10.3174/ajnr.A2530 21546459 PMC7966065

[34] Jiang WJ Cheng-Ching E Abou-Chebl A Zaidat OO Jovin TG Kalia J Multicenter analysis of stenting in symptomatic intracranial atherosclerosis. Neurosurgery (2012) 70:25–30; discussion 31. 10.1227/NEU.0b013e31822d274d 21795866

[35] Schmidt A Piorkowski M Werner M Ulrich M Bausback Y Braunlich S First experience with drug-eluting balloons in infrapopliteal arteries: restenosis rate and clinical outcome. J Am Coll Cardiol (2011) 58:1105–9 10.1016/j.jacc.2011.05.034 21884945

[36] Vajda Z Guthe T Perez MA Heuschmid A Schmid E Bazner H Neurovascular in-stent stenoses: treatment with conventional and drug-eluting balloons. AJNR Am J Neuroradiol (2011) 32:1942–7 10.3174/ajnr.A2644 21885715 PMC7966004

[37] Vajda Z Guthe T Perez MA Kurre W Schmid E Bazner H Prevention of intracranial in-stent restenoses: predilatation with a drug eluting balloon, followed by the deployment of a self-expanding stent. Cardiovasc Intervent Radiol (2013) 36:346–52 10.1007/s00270-012-0450-9 22869043 PMC3595472

